# Comparison of clinical outcomes of intravascular ultrasound-calcified nodule between percutaneous coronary intervention with versus without rotational atherectomy in a propensity-score matched analysis

**DOI:** 10.1371/journal.pone.0241836

**Published:** 2020-11-05

**Authors:** Yusuke Watanabe, Kenichi Sakakura, Yousuke Taniguchi, Kei Yamamoto, Masaru Seguchi, Takunori Tsukui, Hiroyuki Jinnouchi, Hiroshi Wada, Shin-ichi Momomura, Hideo Fujita

**Affiliations:** Division of Cardiovascular Medicine, Saitama Medical Center, Jichi Medical University, Shimotsuke, Japan; Baylor Scott and White, Texas A&M College of Medicine, UNITED STATES

## Abstract

**Objectives:**

This study aimed to compare the mid-term clinical outcomes of intravascular ultrasound (IVUS)-calcified nodules between percutaneous coronary intervention (PCI) with and without rotational atherectomy (RA).

**Background:**

There has been a debate whether to use RA for the revascularization of calcified nodule. Although RA can ablate the calcified structure within calcified nodule and may facilitate adequate stent expansion, RA may provoke severe coronary perforation, because calcified nodule typically shows eccentric calcification.

**Methods:**

We included 204 lesions with IVUS-calcified nodule, and divided into 73 lesions treated with RA (RA group) and 131 lesions without RA (non-RA group). After propensity-score matching, 42 lesions with RA (matched RA group) and 42 lesions without RA (matched non-RA group) were selected. We compared the clinical characteristics and outcomes between the 2 groups before and after propensity-score matching. The primary endpoint was ischemia-driven target vessel revascularization (TVR) within 1 year.

**Results:**

Acute lumen area gain on IVUS was comparable between the matched RA group and matched non-RA group (3.9 ± 2.1 mm^2^ vs. 3.4 ± 1.6 mm^2^, p = 0.18). The stent malapposition at calcified nodules was frequently observed in both groups. The ischemia-driven TVR was not different between the 2 groups before (p = 0.82) and after propensity score-matching (p = 0.87).

**Conclusions:**

The use of RA could not reduce the incidence of ischemia-driven TVR in lesions with IVUS-calcified nodule. Our results do not support the routine use of RA for lesions with IVUS-calcified nodule.

## Introduction

Calcified nodule, which was characterized as eruptive accumulation of small nodular calcification protrude into the lumen, was originally described as a relatively rare cause of sudden cardiac death by Virmani and colleagues [1]. However, most interventional cardiologists did not recognize calcified nodule by coronary angiography alone in the early 2000s, because only postmortem histopathologic studies could show the existence of calcified nodule. After the development of intravascular ultrasound (IVUS), calcified nodule became more familiar to interventional cardiologists, because calcified nodule had distinct IVUS features (irregular and convex luminal surface) [2]. Although calcified nodule are widely recognized in the contemporary PCI [3, 4], the revascularization strategy for calcified nodule has not been established. Although rotational atherectomy (RA) can ablate the calcified structure within calcified nodule and may facilitate adequate stent expansion, RA may provoke severe coronary perforation [5], because calcified nodule typically shows eccentric calcification. On the other hand, if we put a stent over calcified nodule without RA, the stent may not expand sufficiently, because the degree of underlying calcification was closely associated with stent underexpansion [6]. This study aimed to compare the mid-term clinical outcomes of IVUS-calcified nodule between PCI with and without RA.

## Patients and methods

### Study patients

This was a single-center retrospective observational study at Saitama Medical Center, Jichi Medical University. We reviewed the consecutive PCI cases from our hospital records from January 2016 to December 2018. IVUS-calcified nodule were identified by following IVUS criteria: 1) a convex shape of the luminal surface, 2) a convex shape of the luminal side of calcium, 3) an irregular luminal surface, and 4) an irregular leading edge of calcium ^2^. The study inclusion criteria were (1) moderate to severe calcification on coronary angiography, (2) stent implantation was undergone to the lesion with calcified nodule, and (3) IVUS was used before and after stent implantation. The exclusion criteria were (1) none to mild calcification on coronary angiography, (2) in-stent lesion, (3) bypass graft lesions, (4) IVUS images were not adequate (poor IVUS images), (5) the lesions that underwent orbital atherectomy. In analysis 1, we divided our study cases into the lesions that was treated with RA (RA group) and the lesions that was treated without RA (Non-RA group). Clinical characteristics and outcomes were compared between the RA group and non-RA group. Propensity score matching was performed to match the RA group and the non-RA group for variables with a marginal difference (p <0.2) between the 2 groups before propensity score matching. The details of propensity score matching are described in the statistical analysis section. One-to-one propensity score matching resulted in 42 lesions in the matched RA group and 42 lesions in the matched non-RA group. In analysis 2, clinical characteristics and outcomes were compared between the matched RA group and the matched non-RA group. The primary endpoint was defined as ischemia-driven target vessel revascularization (TVR) within 1 year after the index PCI. Ischemia-driven TVR was defined as any revascularization procedure of the target vessel prompted by symptoms or objective evidence of ischemia [7]. The secondary endpoints were non-fatal myocardial infarction and cardiac death within 1 year after the index PCI. The day of PCI to a lesion with calcified nodule was defined as the index day (Day 0). This study was approved by the institutional review board of Saitama Medical Center, Jichi Medical University (S19-138), and written informed consent was waived because of the retrospective study design.

### Definition

**Fig 1:**
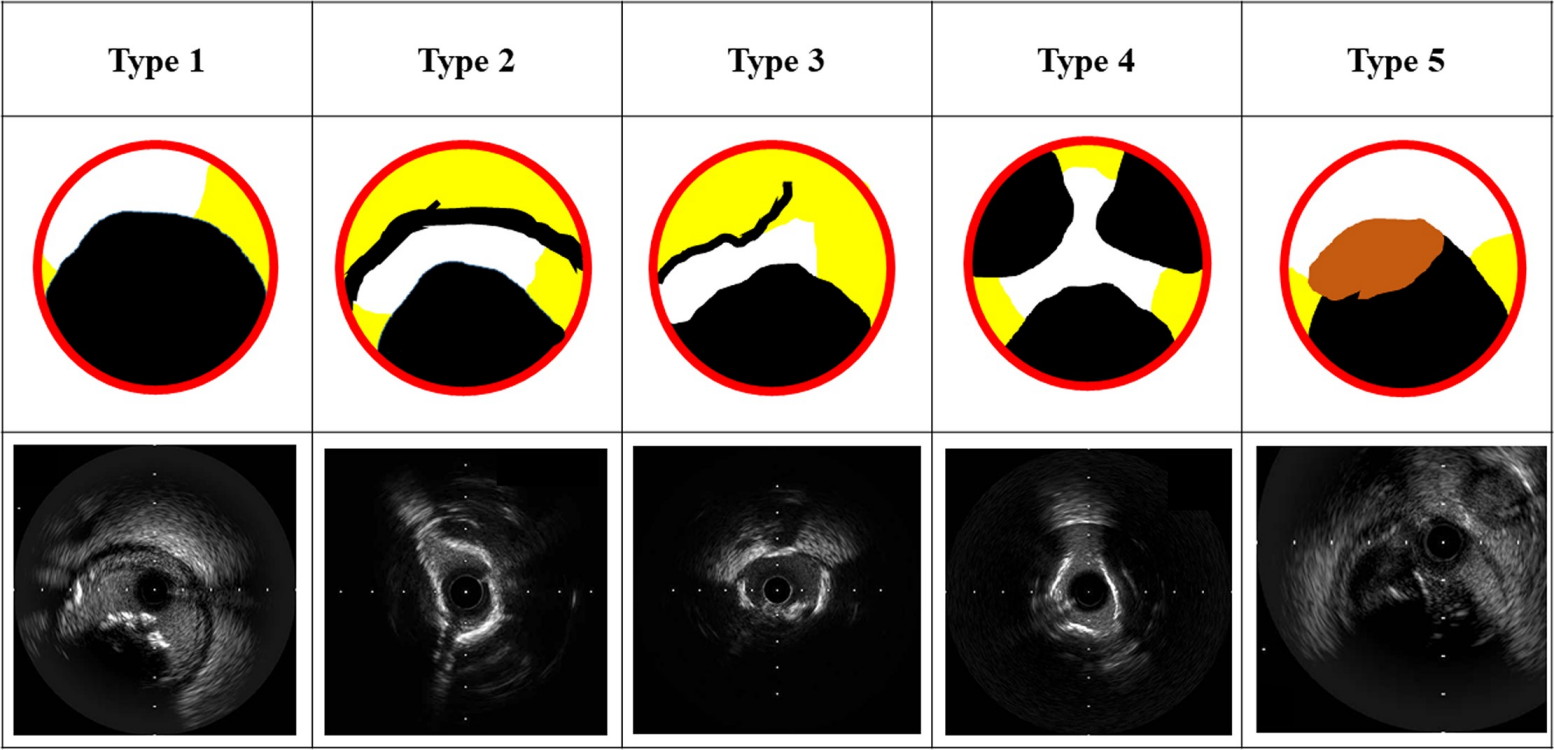
The classification of IVUS-calcified nodules. Upper panels show the schemes of calcified nodules, and lower panels show the corresponding IVUS images. Type 1 was an eccentric calcified nodule without calcification at the opposite site of calcified nodule. Type 2 was an eccentric calcified nodule with broad (≥180° arc) superficial calcification at the opposite site of calcified nodule. Type 3 was an eccentric calcified nodule with narrow (<180° arc) superficial calcification pattern at the opposite site of calcified nodule. Type 4 was multiple calcified nodules within the lumen. Type 5 was a calcified nodule with visible luminal thrombus. Black represents calcification, yellow represents non-calcified plaques, orange represents visible thrombus, white represents vessel lumen, and red represents vessel wall (media).

IVUS-calcified nodules were further classified as type 1, 2, 3, 4 and 5 according to the IVUS findings (Fig 1). Type 1 was defined as an eccentric calcified nodule without superficial calcification at the opposite site of calcified nodule. Type 2 was defined as an eccentric calcified nodule with broad (≥180° arc) superficial calcification at the opposite site of calcified nodule. Type 3 was defined as an eccentric calcified nodule with narrow (<180° arc) superficial calcification pattern at the opposite site of calcified nodule. Type 4 was defined as multiple calcified nodules within the lumen. Type 5 was defined as calcified nodule with visible luminal thrombus. Hypertension was defined as medical treatment for hypertension and/or a history of hypertension before admission [8]. Dyslipidemia was defined as total cholesterol level ≥ 220 mg/dl or low-density lipoprotein cholesterol level ≥ 140 mg/dl or medical treatment for dyslipidemia or a history of dyslipidemia [8]. Diabetes mellitus was defined as hemoglobin A1c level ≥ 6.5% (as NGSP value) or medical treatment for diabetes mellitus or a history of diabetes mellitus [8]. Valvular disease was defined as severe valvular dysfunction or a history of operation. We also calculated estimated glomerular filtration rate (eGFR) from the serum creatinine level, age, weight, and gender using the following formula; eGFR = 194×Cr^-1.094^×age^-0.287^ (male), eGFR = 194×Cr^-1.094^×age^-0.287^×0.739 (female) [9]. Acute myocardial infarction (AMI) was defined according to the fourth universal definition of myocardial infarction [10]. The diagnosis of AMI required the following criteria: symptom consistent with AMI, elevated cardiac enzyme including Troponin T, Troponin I, and/or creatinine kinase (at least 2-folds increase from normal upper limit), and ST-T segment change in electrocardiograms compatible with AMI. Thrombus on angiography was defined as TIMI thrombus grade ≥2 [11]. From coronary angiogram, the reference diameter, lesion length, and minimum diameter were calculated by quantitative coronary angiographic analysis (QCA). Offline, computer-based software QAngio XA 7.3 (MEDIS Imaging Systems, Leiden, Netherlands) was used for QCA analysis. The lesion diameter, external elastic membrane area, lumen area and plaque area at lesion were acquired from IVUS analysis. Qualitative and quantitative analysis of grayscale IVUS images were performed according to the criteria of the American College of Cardiology’s Clinical Expert Consensus Document on IVUS [12]. Complications such as transient slow flow, periprocedural myocardial infarction, and perforation were recorded. Periprocedural myocardial infarction was defined as an increase in creatine kinase (at least a threefold increase above the normal upper limit) [13].

### Statistical analysis

Data are expressed as mean ± standard deviation (S.D.) or percentage. Categorical variables are presented as numbers (percentage) and compared with a Fisher’s exact test. The Kolmogorov–Smirnov test was performed to determine if the continuous variables were normally distributed. Normally distributed continuous variables were compared between the groups using an unpaired Student t test. Otherwise, continuous variables were compared using a Mann–Whitney U-test. One-to-one propensity score matching was used to match the clinical background between the 2 groups. Female sex, hypertension, dyslipidemia, chronic hemodialysis, past medical history of myocardial infarction, left ventricular ejection fraction (LVEF), PCI indication as acute myocardial infarction, temporary pacemaker support, intra-aortic balloon pumping support, calcified nodule at lesion of left circumflex artery, ostium lesion, initial TIMI flow grade 3, minimum lesion diameter on QCA, lesion length on QCA were set as independent variables which had a marginal difference (p < 0.2) between the 2 groups before propensity score matching. For matching, the match tolerance was set as a width of 0.25 multiplied by the S.D. of the propensity score distribution [14, 15]. Incidences of mid-term outcomes after the index PCI were estimated by the Kaplan-Meier method, and the difference between the 2 groups were assessed by the log-rank test. We also compared ischemia-driven TVR within 1 year between the groups using 3 Cox hazard models. In the Model 1, the use of RA, the culprit lesion of STEMI, and lesion length (per 10mm) were included as independent variables. In the Model 2, the use of RA, type 5 of calcified nodule, and lesion length on QCA (per 10mm) were included as independent variables. In the Model 3, the use of RA, lesion EEM area (per 1 mm), and lesion length (per 10mm) were included as independent variables. A P value <0.05 was considered statistically significant. All analyses were performed using statistical software, SPSS 23.0/Windows (SPSS, Chicago, IL).

## Results

**Fig 2:**
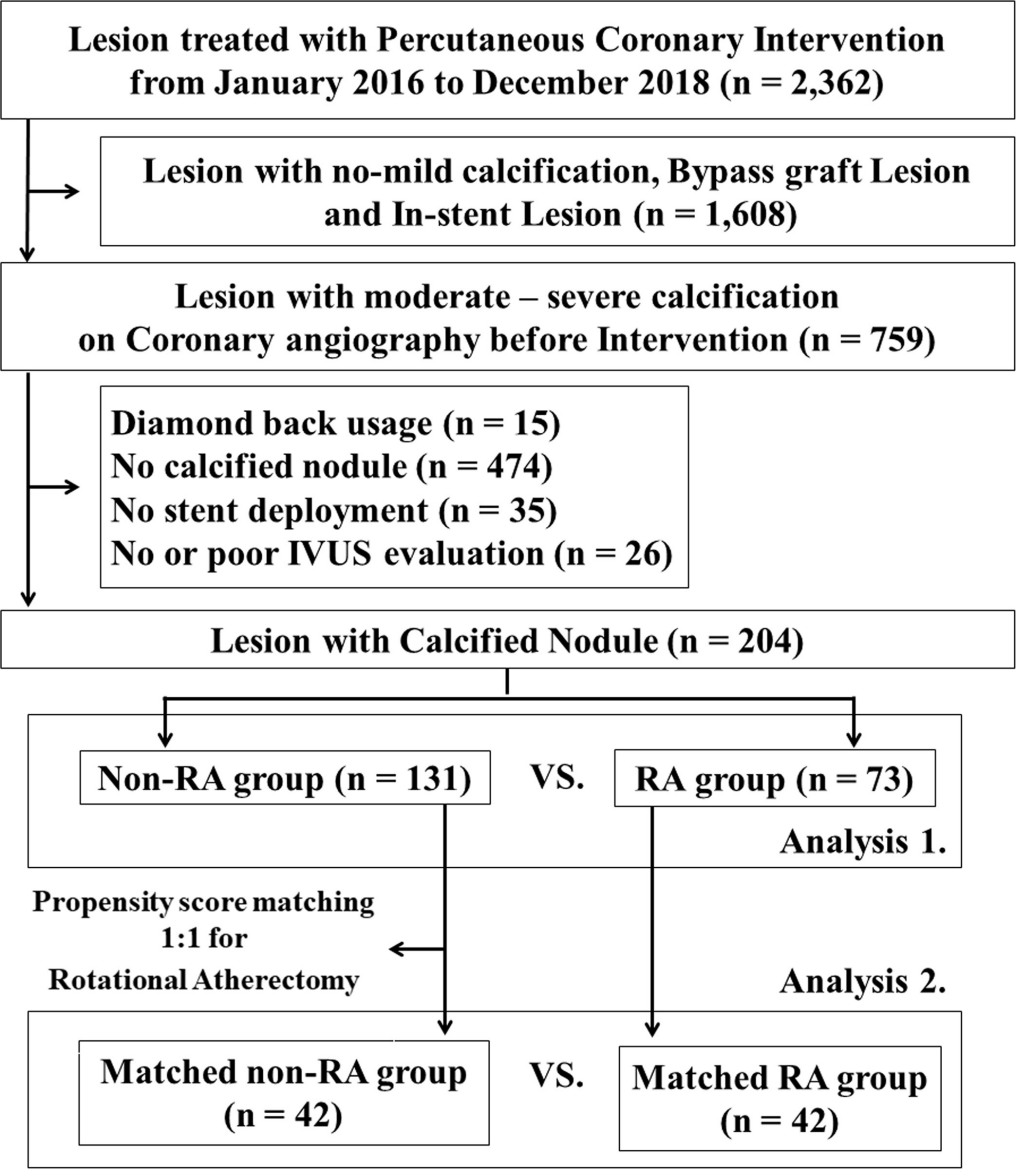
Study flow chart. IVUS = Intravascular ultrasound, RA = rotational atherectomy.

Among 2,362 lesions required PCI in our hospital from January 2016 to December 2018, a total of 204 lesions (73 in the RA group and 131 in the Non-RA group) were included as the study population. After propensity score matching, study population were divided into the matched RA group (n = 42) and the matched non-RA group (n = 42) (Fig 2). During the study period, 251 lesions underwent RA, and only 3 lesions with IVUS-calcified nodule did not receive stent implantation after RA.

Table 1 shows the comparison of patient characteristics between the 2 groups before and after propensity score matching. The prevalence of hypertension and dyslipidemia were significantly greater in the RA group than in the non-RA group, respectively (97.3% vs. 87.8%, p = 0.036; 91.8% vs. 80.9%, p = 0.043). The prevalence of the culprit lesion of STEMI was significantly greater in the non-RA group than in the RA group (21.4% vs. 1.4%, p <0.001), but that was comparable between the matched RA group and the matched non-RA group (9.5% vs. 2.4%, p = 0.49). The degree of LVEF was significantly better in the RA group than in the non-RA group (56.5 ± 14.7% vs. 50.0 ± 14.7%, p < 0.01). Temporary pacemaker was more frequently used in the RA group as compared to the non-RA group (3.1% vs. 34.2%, p = 0.001). All clinical characteristics were comparable between the matched RA group and matched non-RA group. Table 2 shows the comparison of pre-procedural angiographic and IVUS findings between the 2 groups. The grade of initial TIMI flow was significantly better in the RA group than non-RA group (p = 0.004). IVUS-calcified nodule classification was significantly different between the RA group and non-RA group, but was comparable between the matched RA group and the matched non-RA group after propensity-score matching. The other findings between the matched RA group and the matched non-RA group were also comparable.

**Table 1:** Comparison of baseline characteristics between the 2 groups before and after propensity-score matching.

	All patients			Propensity score matched patients		
	Non-RA group (n = 131)	RA group (n = 73)	P value	Non-RA group (n = 42)	RA group (n = 42)	P value
Age, years	73.1 ± 10.0	73.4 ± 9.2	0.72	71.2 ± 10.0	73.6 ± 9.0	0.15
Female sex	28 (21.4)	24 (32.9)	0.093	11 (26.2)	12 (28.6)	1.00
Body mass index, kg/m2	23.7 ± 4.6 (n = 130)	23.2 ± 3.8	0.35	24.4 ± 6.0	23.2 ± 3.4	0.22
Hypertension	115 (87.8)	71 (97.3)	0.036	40 (95.2)	41 (97.6)	1.00
Dyslipidemia	106 (80.9)	67 (91.8)	0.043	36 (85.7)	37 (88.1)	1.00
Diabetes mellitus	67 (51.1)	39 (53.4)	0.76	21 (50.0)	23 (54.8)	0.83
eGFR < 60mL/min/1.73m^2^	46 (63.0)	84 (64.1)	0.88	39.2 ± 30.8	43.9 ± 29.0	0.40
Hemodialysis,	27 (20.6)	22 (30.1)	0.17	14 (33.3)	13 (31.0)	1.00
Peripheral artery disease	37/120 (30.8)	26/70 (37.1)	0.43	17/40 (42.5)	17/40 (42.5)	1.00
Past medical history of myocardial infarction	41 (31.3)	14 (19.2)	0.071	12 (28.6)	11 (26.2)	1.00
Past medical history of cardiac surgery	13 (9.9)	7 (9.6)	1.00	6 (14.3)	5 (11.9)	1.00
Past medical history of cerebrovascular disease	29 (22.1)	18 (24.7)	0.73	11 (26.2)	10 (23.8)	1.00
Left ventricular ejection fraction	50.0± 14.8 (n = 128)	56.5 ± 14.7 (n = 72)	0.001	54.3 ± 13.9	54.8 ± 13.6	0.83
Valvular heart disease	7/129 (5.4)	7/72 (9.7)	0.26	3 (7.1)	3 (7.1)	1.00
Reasons for PCI			<0.001			0.49
ST segment elevation myocardial infarction	28 (21.4)	1 (1.4)		4 (9.5)	1 (2.4)	
Non-ST segment elevation myocardial infarction	38 (29.0)	16 (19.3)		11 (26.2)	11 (26.2)	
Non-acute myocardial infarction	65 (49.6)	56 (76.7)		27 (64.3)	30 (71.4)	
Temporary pacemaker support	4 (3.1)	25 (34.2)	<0.001	4 (9.5)	4 (9.5)	1.00
Intra-aortic balloon pumping support	10 (7.6)	11 (15.1)	0.15	3 (7.1)	3 (7.1)	1.00
V-A ECMO support	3 (2.3)	1 (1.4)	1.00	0 (0.0)	0 (0.0)	N/A

Categorical variables are expressed as number and (%). Continuous variables are indicated as mean ± SD.

PCI = percutaneous coronary intervention; V-A ECMO = veno-arterial extracorporeal membrane oxygenation.

**Table 2:** Pre-procedural angiographic and IVUS findings between the 2 groups before and after propensity-score matching.

	All patients			Propensity score matched patients		
	Non-RA group (n = 131)	RA group (n = 73)	P value	Non-RA group (n = 42)	RA group (n = 42)	P value
**Angiographic findings**						
Lesion details with calcified nodule			0.15			0.12
Right coronary artery	37 (28.2)	23 (31.5)		15 (35.7)	6 (14.3)	
Left main coronary trunk	6 (4.6)	5 (6.8)		2 (4.8)	4 (9.5)	
Left anterior descending artery	70 (53.4)	42 (575)		24 (57.1)	30 (71.4)	
Left circumflex artery	18 (13.7)	3 (4.1)		1 (2.4)	2 (4.8)	
Ostium lesion	7 (5.3)	9 (12.3)	0.10	2 (4.8)	4 (9.5)	0.68
Thrombus	14 (10.7)	2 (2.7)	0.056	1 (2.4)	2 (4.8)	1.00
Initial TIMI grade			0.004			0.31
grade 0	15 (11.5)	0 (0.0)		3 (7.1)	0 (0.0)	
grade 1	8 (6.1)	2 (3.6)		3 (7.1)	1 (2.4)	
grade 2	35 (26.7)	19 (26.0)		9 (21.4)	11 (26.2)	
grade 3	73 (55.7)	52 (71.2)		27 (64.3)	30 (71.4)	
**QCA findings before stent deployment**						
Reference diameter, mm	2.5 ± 0.7	2.6 ± 0.6	0.27	2.6 ± 0.6	2.6 ± 0.6	0.89
Minimum lesion diameter, mm	0.7 ± 0.4	0.9 ± 0.4	0.005	0.8 ± 0.3	0.8 ± 0.3	0.59
Lesion length, mm	19.4 ± 14.4	23.0 ± 14.6	0.052	26.3 ± 19.6	22.5 ± 15.3	0.53
**IVUS findings before stent deployment**						
Lesion external elastic membrane area, mm^2^	14.1 ± 4.2	14.7 ± 4.8	0.42	14.3 ± 3.9	14.5 ± 4.8	0.85
Lesion lumen area, mm^2^	2.6 ± 0.9	3.0 ± 1.1	0.008	2.9 ± 1.2	3.1 ± 1.0	0.52
Lesion plaque area, mm^2^	11.5 ± 3.9	11.7 ± 4.3	0.71	11.4 ± 3.5	11.4 ± 4.4	0.98
**IVUS-Calcified Nodule classification**			<0.001			0.18
Type 1	49 (37.4)	32 (43.8)		20 (47.6)	18 (42.9)	
Type 2	14 (10.7)	14 (19.2)		2 (4.8)	8 (19.0)	
Type 3	21 (16.0)	14 (19.2)		8 (19.0)	8 (19.0)	
Type 4	13 (9.9)	11 (15.1)		7 (16.7)	7 (16.7)	
Type 5	34 (26.0)	2 (2.7)		5 (11.9)	1 (2.4)	

Categorical variables are expressed as number and (%). Continuous variables are indicated as mean ± SD.

IVUS = intravascular ultrasound, QCA = quantitative coronary angiographic analysis, TIMI = Thrombolysis in Myocardial infarction

Table 3 shows the comparison of procedural and post-procedural angiographic and IVUS findings between the 2 groups. The location of dissection/crack after balloon dilatation with/without RA was mainly the sides of calcified nodules (S1 Fig), while the complete breakdown of the calcified nodule itself was never seen in this study. The minimum lesion diameter after stent deployment on QCA and lesion lumen area after stent deployment on IVUS were significantly greater in the RA group than in the non-RA group, but these were not significantly different between the matched RA group and the matched non-RA group. Acute lumen area gain was comparable between the matched RA group and matched non-RA group. Acute lumen area gain was also comparable among 5 types of IVUS-calcified nodule (S1 Table). Furthermore, the stent malapposition at calcified nodules was frequently observed in both groups.

**Table 3:** Procedural and post-procedural angiographic and IVUS findings between the 2 groups before and after propensity-score matching.

	All patients			Propensity score matched patients		
	Non-RA group (n = 131)	RA group (n = 73)	P value	Non-RA group (n = 42)	RA group (n = 42)	P value
Type of pre-dilatation balloon			<0.001			0.048
Semi-compliant balloon	46 (35.1)	31 (42.5)		14 833.3)	19 (45.2)	
Non-compliant balloon	28 (21.4)	1 (1.4)		8 (19.0)	1 (2.4)	
Scoring balloon	25 (19.1)	27 (37.0)		12 (28.6)	17 (40.5)	
Cutting balloon	0 (0.0)	1 (1.4)		0 (0.0)	0 (0.0)	
≥2 types of balloons	32 (24.4)	13 (17.8)		8 (19.0)	5 (11.9)	
Pre-dilatation diameter, mm	2.42 ± 0.31	2.66 ± 0.28	<0.001	2.52 ± 0.30	2.67 ± 0.28	0.037
Pre-dilatation max pressure, atm	16.4 ± 4.1	17.2 ± 3.8	0.21	16.5 ± 4.2	17.3 ± 4.0	0.36
Use of additional balloon for post-dilatation	87 (66.4)	46 (63.0)	0.65	21 (50.0)	16 (38.1)	0.27
Post-dilatation balloon diameter, mm	3.46 ± 0.64 (n = 87)	3.64± 0.70 (n = 46)	0.17	3.43 ± 0.51 (n = 21)	3.23 ± 0.57 (n = 16)	0.25
Post-dilatation balloon max pressure, atm	17.5 ± 3.6 (n = 87)	17.4± 3.3 (n = 46)	0.88	18.5 ± 2.8 (n = 21)	19.2 ± 3.2 (n = 16)	0.50
Number of burrs used	-	1.2 ± 0.5	-	-	1.2 ± 0.5	-
Initial burr size	-					-
1.25-mm	-	36 (49.3)	-	-	22 (52.4)	-
1.5-mm	-	37 (50.7)	-	-	20 (47.6)	-
Final burr size	-					-
1.25-mm	-	31 (42.5)	-	-	20 (47.6)	-
1.5-mm	-	31 (42.5)	-	-	16 (38.1)	-
1.75-mm	-	3 (4.1)	-	-	0 (0.0)	-
2.0-mm	-	8 (11.0)	-	-	6 (14.3)	-
Initial burr-to-artery ratio	-	0.56 ± 0.14	-	-	0.56 ± 0.14	-
Final burr-to-artery ratio	-	0.59 ± 0.15	-	-	0.59 ± 0.14	-
**Type of Stent deployed to calcified nodule**			0.085			0.58
Covered stent	0 (0.0)	1 (1.4)		0 (0.0)	0 (0.0)	
Bare metal stent	3 (2.3)	3 (4.1)		2 (4.8)	1 (2.4)	
Durable polymer Everolimus Eluting Stent (Promus)	0 (0.0)	1 (1.4)		0 (0.0)	1 (2.4)	
Durable polymer Everolimus Eluting Stent (Xience)	30 (22.9)	12 (16.4)		9 (21.4)	5 (11.9)	
Durable polymer Zotarolimus Eluting Stent (Resolute)	40 (30.5)	13 (17.8)		11 (26.2)	8 (19.0)	
Biodegradable polymer Everolimus Stent (Synergy)	48 (36.6)	36 (49.3)		15 (35.7)	21 (50.0)	
Biodegradable polymer Sirolimus Stent (Ultimaster)	10 (7.6)	7 (9.6)		5 (11.9)	6 (14.3)	
Stent diameter, mm	2.74 ± 0.37	2.91 ± 0.42	0.008	2.85 ± 0.37	2.84 ± 0.41	0.77
Stent length, mm	26.5 ± 8.0	28.5 ± 9.0	0.071	26.4 ± 8.0	27.7 ± 9.3	0.45
**Final TIMI flow grade**			N/A			N/A
grade 3	131 (100)	73 (100)		42 (100)	42 (100)	
**QCA after stent deployment**						
Reference diameter, mm	3.0 ± 0.5	3.2 ± 0.5	<0.001	3.1 ± 0.6	3.1 ± 0.5	0.46
Minimum lesion diameter, mm	2.6 ± 0.4	2.9 ± 0.5	<0.001	2.8 ± 0.5	2.8 ± 0.4	0.66
Acute lumen gain (Final minimum lesion diameter–initial minimum lesion diameter)	2.0 ± 0.5	2.0 ± 0.5	0.34	2.0 ± 0.6	2.0 ± 0.4	0.82
**IVUS findings after balloon dilatation ± rotational atherectomy**						
Dissection before stent deployment	36 (27.6)	26 (35.6)	0.27	15 (35.7)	14 (33.3)	1.00
Details of dissection and/or crack before stent deployment			0.69			1.00
None	95 (72.5)	47 (64.4)		27 (64.3)	28 (66.7)	
Sides of calcified nodule	24 (18.3)	18 (24.7)		9 (21.4)	9 (21.4)	
Superficial calcification at the other site of calcified nodule	8 (6.1)	6 (8.2)		4 (9.5)	4 (9.5)	
Sides of calcified nodule, and superficial calcification at the other site of calcified nodule	3 (2.3)	2 (2.7)		1 (2.4)	1 (2.4)	
Plaque at the other site of calcified nodule	1 (0.8)	0. (0.0)		1 (1.4)	0 (0.0)	
**IVUS findings after stent deployment**						
Lesion lumen area, mm^2^	6.0 ± 1.7	6.9 ± 2.1	0.005	6.8 ± 2.0	6.5 ± 1.8	0.43
Lumen area gain (final–pre lesion lumen area), mm2	3.4 ± 1.7	3.9 ± 1.8	0.15	3.9 ± 2.1	3.4 ± 1.6	0.18
Proximal stent edge mal-apposition	62 (47.3)	35 (47.9)	1.00	20 (47.6)	21 (50.0)	1.00
Distal stent edge mal-apposition	22/119 (18.5)	15/65 (23.1)	0.45	7/38 (18.4)	9/38 (23.7)	0.78
In-stent mal-apposition (at calcified nodule)	70 (53.4)	49 (67.1)	0.075	23 (54.8)	31 (73.8)	0.11
In-stent mal-apposition (at lesion without calcified nodule)	74 (56.5)	53 (72.6)	0.025	24 (57.1)	31 (73.8)	0.17
Proximal/distal stent edge dissection	0 (0.0)	0 (0.0)	N/A	0 (0.0)	0 (0.0)	N/A
**Complications**						
Transient slow flow	11 (8.4)	10 (13.7)	0.24	2 (4.8)	5 (11.9)	0.43
Perforation at the time of stent implantation	0 (0.0)	1 (1.4)	0.36	0 (0.0)	0 (0.0)	N/A
Periprocedural myocardial infarction	26 (19.8)	2 (2.7)	<0.001	5 (11.9)	2 (4.8)	0.43

Categorical variables are expressed as number and (%). Continuous variables are indicated as mean ± SD.

IVUS = intravascular ultrasound, QCA = quantitative coronary angiographic analysis, TIMI = Thrombolysis in Myocardial infarction

**Fig 3:**
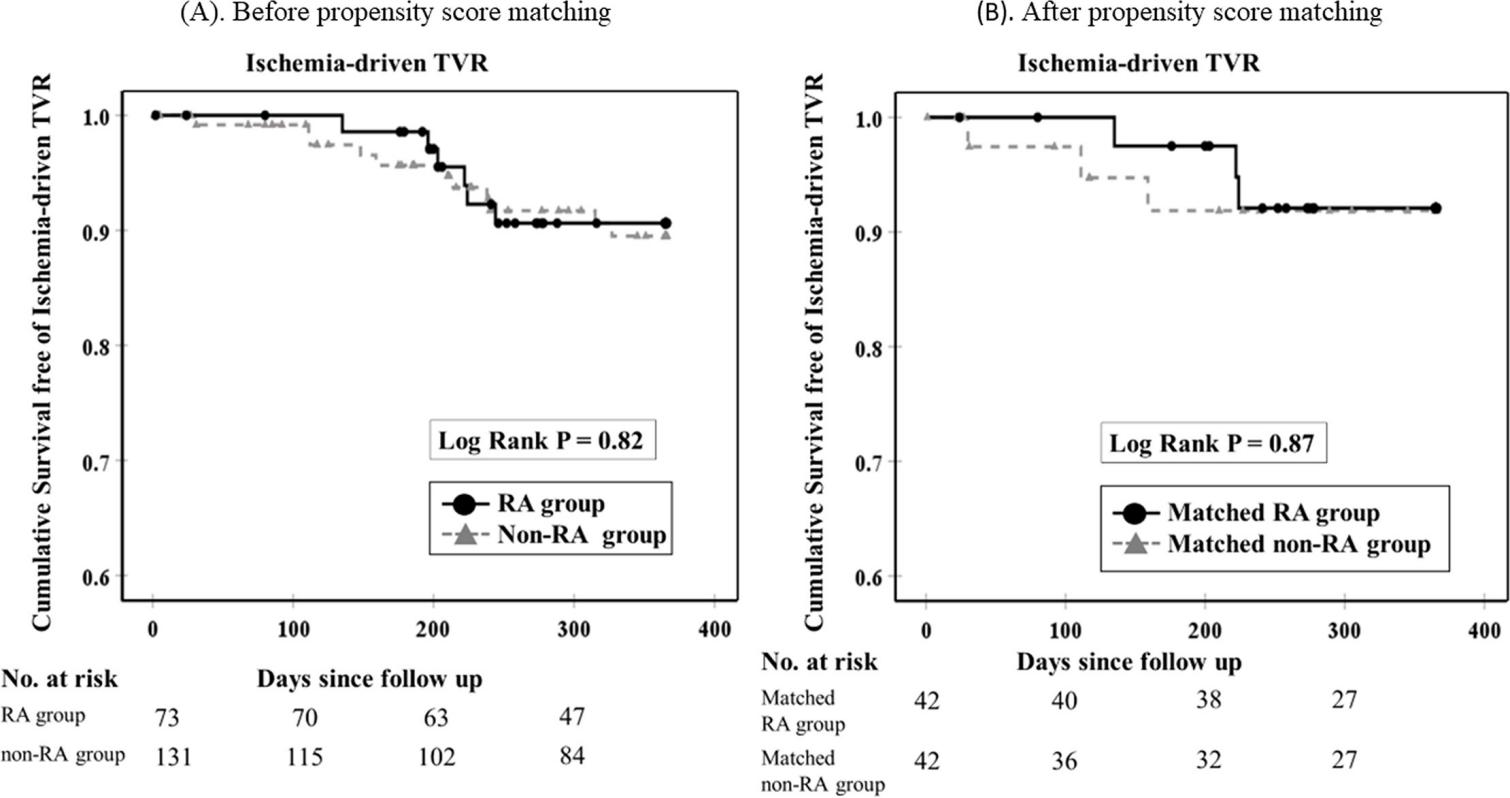
Kaplan-Meier curves of cumulative survival free of ischemia-driven TVR events within one year between two groups before and after propensity score matching. Survival curves of ischemia-driven TVR are shown for the non-RA and the RA groups, and for the matched non-RA group and the matched RA group. A log-rank test showed no significant difference between the two groups in before (p = 0.82) and after propensity-score matching (p = 0.87).

Fig 3 shows ischemia-driven TVR free survival curves between the 2 groups. During the follow-up period, a total of 6 ischemia-driven TVR were observed in the RA group, whereas 6 ischemia-driven TVR were observed in the non-RA group. Ischemia-driven TVR free survival curves were not different between the 2 groups before (p = 0.82) and after (p = 0.87) propensity score matching. Non-fatal myocardial infarction free survival curves were not different between the 2 groups before and after propensity score matching (S2 Fig). Furthermore, cardiac death free survival curves were not different between the 2 groups before propensity score matching (S3 Fig). There was no cardiac death in both groups after propensity score matching. Table 4 shows the multivariate Cox hazard models to find association with ischemia-driven TVR before and after propensity score matching. The use of RA was not associated with the incidence of ischemia-driven TVR in any models before and after propensity score matching.

**Table 4:** Multivariate Cox hazard models to find associations with ischemia-driven TVR before and after propensity score matching.

	Before Propensity-score matching			After Propensity-score matching		
	HR	95% CI	P value	HR	95% CI	P value
**Model 1.**						
Use of rotational atherectomy to calcified nodule	0.733	0.265–2.025	0.55	0.915	0.180–4.658	0.92
ST segment elevation myocardial infarction as onset	0.395	0.051–3.062	0.37	N/A	N/A	N/A
Lesion length on QCA (per 10mm)	1.177	0.897–1.544	0.42	1.217	0.825–1.795	0.32
**Model 2.**						
Use of rotational atherectomy to calcified nodule	0.747	0.267–2.094	0.58	0.868	0.172–4.389	0.87
Calcified nodule type 5	0.604	0.132–2.757	0.52	N/A	N/A	N/A
Lesion length on QCA (per 10mm)	1.178	0.896–1.550	0.24	1.229	0.836–1.808	0.29
**Model 3.**						
Use of rotational atherectomy to calcified nodule	0.790	0.290–2.151	0.64	0.962	0.188–4.933	0.96
Lesion external elastic membrane area on IVUS (per 1 mm)	1.069	0.968–1.181	0.19	1.080	0.916–1.272	0.36
Lesion length on QCA (per 10mm)	1.184	0.901–1.555	0.23	1.230	0.834–1.816	0.30

IVUS = intravascular ultrasound, QCA = quantitative coronary angiographic analysis, HR = hazard ratio, CI = confidence interval.

## Discussion

We included 204 lesions with IVUS-calcified nodule, and divided into the RA group (n = 73) and the non-RA group (n = 131). After propensity score matching, the matched RA group (n = 42) and the matched non-RA group (n = 42) were generated from the RA group and the non-RA group, respectively. The main findings of this study were as follows: 1) The primary endpoint (ischemia-driven TVR) was not different between the RA group and non-RA group before and after propensity score matching. Furthermore, the multivariate Cox hazard models also confirmed that there was no significant association between the incidence of ischemia-driven TVR and the use of RA after controlling covariates. 2) acute lumen area gain was similar between the 2 groups before and after propensity score matching, suggesting the limited effect of RA. 3) The most dominant type of calcified nodule was type 1, which was defined as an eccentric calcified nodule without calcification at the opposite site of calcified nodule.

In the present study, the ischemia-driven TVR following PCI to calcified nodule was not different between lesions with and without RA. In the ROTAXUS study, although the use of RA was associated with the greater acute lumen gain, the use of RA could not decrease either late lumen loss or target lesion revascularization [16]. Similarly, in the randomized PREPARE-CALC trial, either mid-term TVR or late lumen loss was not different between the use of modified balloon and use of RA for calcified lesions [17]. Both the ROTAXUS and PREPARE-CALC studies could not show the favorable results regarding late lumen loss, but showed the greater initial procedural success as compared to PCI without RA [16, 17], which suggests that the routine use of RA would not be necessary for severely calcified lesions, but some specific calcified lesions would require RA for an initial procedural success. Since those previous studies did not provide intravascular imaging findings regarding calcified lesions, we focused on calcified nodule detected by IVUS. Before conducting the present study, we speculated that RA would decrease the calcified plaques and may reduce malapposed struts, which would justify the short dual antiplatelet therapy following DES implantation. However, our results revealed that the incidence of in-stent malapposition was greater in the RA group than in the non-RA group without reaching statistical analysis. Thus, our speculation was not proved in this study. Furthermore, PCI with RA could not show the advantage regarding ischemia-driven TVR over PCI without RA. Our results would not support the routine use of RA to lesions with calcified nodule.

We should discuss the reasons why there was no significant difference of ischemia-driven TVR between the RA and non-RA groups. A possible explanation is that acute lumen area gain was similar between the 2 groups before and after the propensity score matching. Our detailed IVUS analysis also showed that the location of dissection and/or crack was similar between the RA and non-RA groups, and that the complete breakdown of the calcified nodule was not observed either in the RA group or in the non-RA group. Our IVUS findings (no significant crack on the top of calcified nodule) is consistent to a dedicated optical coherence tomography study, which showed that calcium cracks after angioplasty was observed in calcified lesions with a larger calcium arc (median 360°, IQR 246-360°) or a thinner calcium thickness (0.53 ± 0.28mm) [18]. Because most of calcified nodules were eccentric and thick calcified lesions, it should be difficult to make a crack on the top of calcified nodule. Moreover, the most dominant type of IVUS-calcified nodules in the present study was an eccentric calcified nodule without calcification at the opposite site of the calcified nodule (Type 1). Normal vessel structure at the opposite site of the IVUS-calcified nodule might allow adequate stent expansion even in the non-RA group.

Clinical implications of the present study should be noted. Since initial acute lumen area gain and mid-term ischemia-driven TVR were not different between PCI with and without RA, the routine use of RA to calcified nodule would not be recommended. Aggressive RA would not result in the elimination of calcified nodules. Conventional balloon dilatation may work for the lesions with calcified nodules, because the most typical type of calcified nodules was an eccentric nodule without calcification at the opposite site of calcified nodule. However, we should mention that our retrospective study would have a significant clinical bias regarding the use of RA. Because of the difficulty in device delivery, some lesions required RA for an initial procedural success. Therefore, RA was definitely necessary in some calcified nodules. On the other hand, since there were no complete breakdowns/cracks at the top of calcified nodules in the present study, the benefit of RA with big RA burrs may be limited. The big burr may damage the normal vessel structure at the opposite site of calcified nodules without making a crack on calcified nodules. Although there were some inherent limitations regarding retrospective design, this propensity score matching analysis could provide an insight into the debate whether to use RA for calcified nodules.

### Study limitation

As we mentioned in the clinical implications, there is a risk of selection bias. Second, we included calcified nodules defined by IVUS features (irregular and convex luminal surface) according to the previous study [2], which would include nodular calcification as well as calcified nodule. Only our type 5 calcified nodule (calcified nodule with luminal thrombus) might correspond to the calcified nodule defined by human pathological studies [1], whereas our type 1 to 4 calcified nodules might correspond to the nodular calcification. However, since the resolution of IVUS was not satisfactory to discriminate small thrombus around nodule, our type 1 to 4 calcified nodules also might include real calcified nodules. It would be difficult for IVUS to discriminate calcified nodules from nodular calcifications. Therefore, we used the word “IVUS-calcified nodule” for this study. Third, we tried to avoid using RA in primary PCI, because RA in primary PCI was significantly associated with adverse events according to the J-PCI registry findings [19]. Such clinical background could be a bias whether to use RA for lesions with calcified nodule.

## Conclusions

The use of RA could not reduce the incidence of ischemia-driven TVR in lesions with IVUS-calcified nodule. Our results do not support the routine use of RA for lesions with calcified nodule.

## Supporting information

S1 Fig The representative image of IVUS-calcified nodule with dissection at the side of calcified nodule. Fig 1A and 1B are same images, whereas Fig 1B has asterisk and arrowhead. This calcified nodule (arrowhead) was treated with rotational atherectomy and balloon dilatation. There was dissection (asterisk) at the side of calcified nodule.
(DOCX)

S2 Fig Kaplan-Meier curves of cumulative survival free of non-fatal myocardial infarction events within one year between two groups before and after propensity score matching. Survival curves of non-fatal myocardial infarction are shown for the non-RA and the RA groups, and for the matched non-RA group and the matched RA group. A log-rank test showed no significant difference between the two groups before (p = 0.56) and after propensity score matching (p = 0.88).
(DOCX)

S3 Fig Kaplan-Meier curves of cumulative survival free of cardiac events within one year between two groups. Survival curves of cardiac death are shown for the non-RA and the RA group. After propensity score matching, no cardiac death event was observed. A log-rank test showed no significant difference between the two groups (p = 0.13).
(DOCX)

S1 Table Detailed analysis of acute lumen area gain with or without rotational atherectomy among 5 types of IVUS-calcified nodule before and after propensity score matching. (DOCX)

S1 Dataset (XLSX)

S2 Dataset (XLSX)
