# Supplementary material for: Comparison of clinical outcomes of intravascular ultrasound-calcified nodule between percutaneous coronary intervention with versus without rotational atherectomy in a propensity-score matched analysis
Source: PLoS One. 2020 Nov 5;15(11):e0241836. doi: 10.1371/journal.pone.0241836 (PMC7643997; doi:10.1371/journal.pone.0241836)
Supplement: S1 Fig — Fig 1A and 1B are same images, whereas Fig 1B has asterisk and arrowhead. This calcified nodule (arrowhead) was treated with rotational atherectomy and balloon dilatation. There was dissection (asterisk) at the side of calcified nodule. (DOCX) [file pone.0241836.s001.docx]

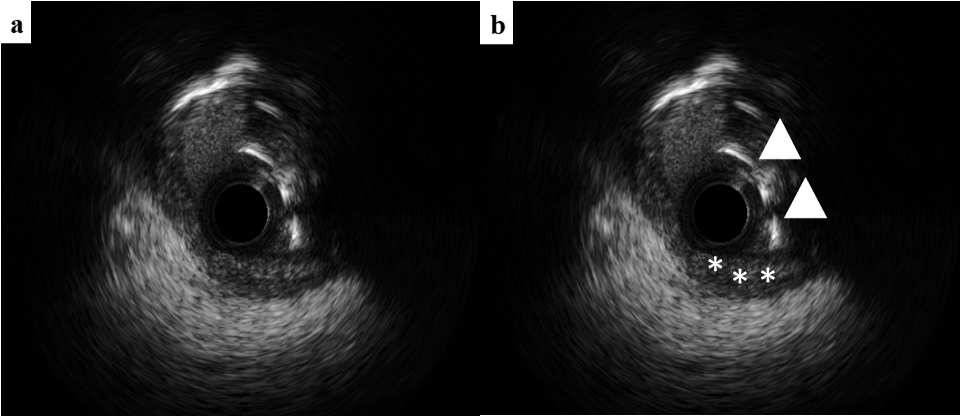


**S1 Fig.** The representative image of IVUS-calcified nodule with dissection at the side of calcified nodule. Figure 1a and 1b are same images, whereas Figure 1b has asterisk and arrowhead. This calcified nodule (arrowhead) was treated with rotational atherectomy and balloon dilatation. There was dissection (asterisk) at the side of calcified nodule.
