# Supplementary material for: Comparison of clinical outcomes of intravascular ultrasound-calcified nodule between percutaneous coronary intervention with versus without rotational atherectomy in a propensity-score matched analysis
Source: PLoS One. 2020 Nov 5;15(11):e0241836. doi: 10.1371/journal.pone.0241836 (PMC7643997; doi:10.1371/journal.pone.0241836)
Supplement: S2 Fig — Survival curves of non-fatal myocardial infarction are shown for the non-RA and the RA groups, and for the matched non-RA group and the matched RA group. A log-rank test showed no significant difference between the two groups before (p = 0.56) and after propensity score matching (p = 0.88). (DOCX) [file pone.0241836.s002.docx]

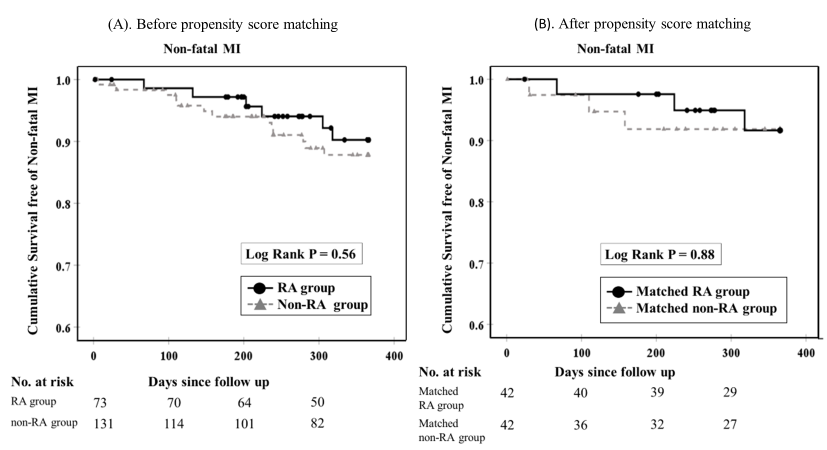


**S2 Fig.** Kaplan-Meier curves of cumulative survival free of non-fatal myocardial infarction events within one year between two groups before and after propensity score matching. Survival curves of non-fatal myocardial infarction are shown for the non-RA and the RA groups, and for the matched non-RA group and the matched RA group. A log-rank test showed no significant difference between the two groups before (p=0.56) and after propensity score matching (p=0.88).
