# Supplementary material for: Comparison of clinical outcomes of intravascular ultrasound-calcified nodule between percutaneous coronary intervention with versus without rotational atherectomy in a propensity-score matched analysis
Source: PLoS One. 2020 Nov 5;15(11):e0241836. doi: 10.1371/journal.pone.0241836 (PMC7643997; doi:10.1371/journal.pone.0241836)
Supplement: S3 Fig — Survival curves of cardiac death are shown for the non-RA and the RA group. After propensity score matching, no cardiac death event was observed. A log-rank test showed no significant difference between the two groups (p = 0.13). (DOCX) [file pone.0241836.s003.docx]

**
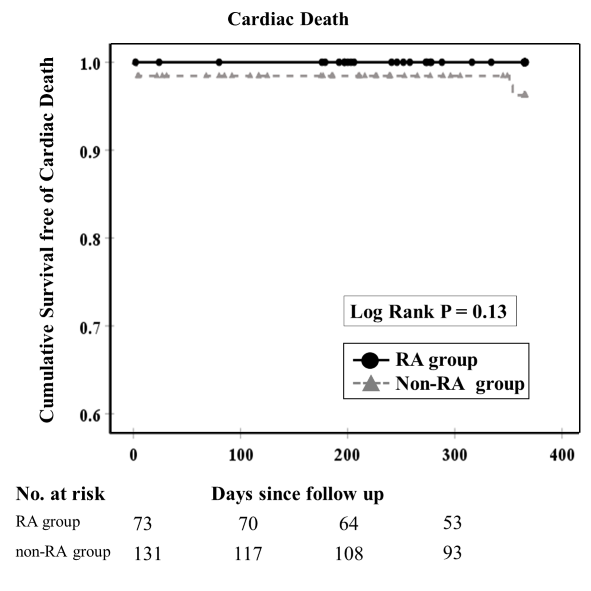
**

**S3 Fig.** Kaplan-Meier curves of cumulative survival free of cardiac events within one year between two groups. Survival curves of cardiac death are shown for the non-RA and the RA group. After propensity score matching, no cardiac death event was observed. A log-rank test showed no significant difference between the two groups (p=0.13).
