# Supplementary material for: Comparison of clinical outcomes of intravascular ultrasound-calcified nodule between percutaneous coronary intervention with versus without rotational atherectomy in a propensity-score matched analysis
Source: PLoS One. 2020 Nov 5;15(11):e0241836. doi: 10.1371/journal.pone.0241836 (PMC7643997; doi:10.1371/journal.pone.0241836)
Supplement: S1 Table — (DOCX) [file pone.0241836.s004.docx]

**S1 Table.** The results of acute lumen gain with or without rotational atherectomy by types of calcified nodule/nodular calcification before and after propensity score matching.

| **Before propensity score matching** | | | | | |  |
| --- | --- | --- | --- | --- | --- | --- |
|  | **Type1** | **Type2** | **Type3** | **Type4** | **Type5** | **P value** |
| Acute lumen gain with RA | 4.18 ± 1.93  (n = 32) | 3.81 ± 2.03  (n = 14) | 3.67 ± 1.81  (n = 14) | 3.38 ± 1.54  (n = 11) | 3.46 ± 1.06  (n = 2) | 0.75 |
| Acute lumen gain without RA | 3.33 ± 1.44  (n = 49) | 3.45±2.16  (n = 12) | 4.01±2.33  (n = 21) | 3.14±1.60  (n = 13) | 3.34 ± 1.49  (n = 34) | 0.88 |
| **After propensity score matching** | | | | | | |
|  | **Type1** | **Type2** | **Type3** | **Type4** | **Type5** | **P value** |
| Acute lumen gain with RA | 4.18 ± 1.93  (n = 32) | 3.81± 2.03  (n = 14) | 3.67 ± 1.81  (n = 14) | 3.38 ± 1.54  (n = 11) | 3.46 ± 1.06  (n = 2) | 0.75 |
| Acute lumen gain without RA | 3.85 ± 1.62  (n = 20) | 4.70 ± 0.32  (n = 2) | 4.82 ± 3.42  (n = 8) | 2.94 ± 1.80  (n = 7) | 3.62 ±1.28  (n = 5) | 0.66 |

Continuous variables are indicated as mean ± SD.

RA = rotational atherectomy.
